# Rituximab therapy achieves comparable remission to cyclophosphamide in ANCA-associated vasculitis with severe kidney involvement

**DOI:** 10.1093/ckj/sfag290

**Published:** 2026-09-03

**Authors:** Nishant Chaudhari, Azm Ul Hussain, Lee Quill, Joshua Harridine, Kashif Eqbal, Lorraine Harper, Dimitrios Chanouzas

**Affiliations:** Renal Unit, Queen Elizabeth Hospital Birmingham, University Hospitals Birmingham NHS Foundation Trust, Birmingham, UK; Renal Unit, Queen Elizabeth Hospital Birmingham, University Hospitals Birmingham NHS Foundation Trust, Birmingham, UK; Department of Immunology and Immunotherapy, College of Medicine and Health, University of Birmingham, Birmingham, UK; Renal Unit, Queen Elizabeth Hospital Birmingham, University Hospitals Birmingham NHS Foundation Trust, Birmingham, UK; Renal Unit, Heartlands Hospital, University Hospitals Birmingham NHS Foundation Trust, Birmingham, UK; Renal Unit, Heartlands Hospital, University Hospitals Birmingham NHS Foundation Trust, Birmingham, UK; Renal Unit, Queen Elizabeth Hospital Birmingham, University Hospitals Birmingham NHS Foundation Trust, Birmingham, UK; Department of Applied Health Sciences, College of Medicine and Health, University of Birmingham, Birmingham, UK; Renal Unit, Queen Elizabeth Hospital Birmingham, University Hospitals Birmingham NHS Foundation Trust, Birmingham, UK; Department of Immunology and Immunotherapy, College of Medicine and Health, University of Birmingham, Birmingham, UK

**Keywords:** ANCA, cyclophosphamide, induction, rituximab, vasculitis

## Abstract

**Background:**

The RAVE (Rituximab versus Cyclophosphamide for ANCA-Associated Vasculitis) and RITUXIVAS (Rituximab versus Cyclophosphamide in ANCA-Associated Renal Vasculitis) trials established rituximab (RTX) therapy in antineutrophil cytoplasmic antibody–associated vasculitis (AAV). However, RAVE excluded patients with severe renal involvement (creatinine >354 µmol/L). Whilst RITUXIVAS did not, treatment included RTX with two cyclophosphamide (CYC) pulses. This study reports outcomes in AAV patients with severe renal involvement treated with RTX alone compared with CYC, together with high-dose steroids.

**Methods:**

Data were collected retrospectively for all consecutive AAV presentations with severe renal involvement (peak creatinine >354 µmol/L) between 2010 and 2023 from two renal units. The primary outcome was sustained remission with independent renal function at 12 months.

**Results:**

Amongst 107 *de novo* AAV patients, 66 received pulsed CYC with steroids [39 received plasma exchange (PLEX)], and 41 received RTX with steroids (7 received PLEX). At presentation, there was no significant difference in renal replacement therapy requirement (CYC 56% vs RTX 71%; *P *= .129), peak creatinine (511 vs 538 µmol/L; *P *= .399) or estimated glomerular filtration rate (eGFR) (9 vs 7 mL/min/1.73 m^2^; *P *= .372). Fifty-two percent of CYC patients and 49% of RTX patients met the primary outcome (*P *= .783). At 12 months, there was no significant difference in the proportion of patients with independent renal function (CYC 65% vs RTX 56%; *P *= .582), eGFR was similar between CYC and RTX patients (28 vs 31 mL/min/1.73 m^2^; *P *= .439), and 94% of the CYC cohort were alive compared with 83% of the RTX cohort (*P *= .068).

**Conclusion:**

RTX with steroids is comparable to CYC with steroids for successfully inducing remission in AAV with severe renal involvement.

KEY LEARNING POINTS **What was known:**

- The RAVE and RITUXIVAS trials previously established rituximab (RTX) as the main induction of remission agent, alongside cyclophosphamide (CYC), in antineutrophil cytoplasmic antibody–associated vasculitis (AAV).
- However, neither study addressed the sole impact of RTX alone compared with CYC in severe renal involvement (defined as creatinine >354 µmol/L at presentation).

**This study adds:**

- This retrospective cohort study reports the outcomes of pulsed CYC with high-dose corticosteroids, compared with RTX with high-dose corticosteroids in a cohort of over 100 patients with AAV and severe renal involvement at presentation.
- Remission outcomes and renal recovery were comparable across RTX- and CYC-treated patients.

**Potential impact:**

- RTX is comparable to CYC in inducing remission in AAV with severe renal impairment.
- Future studies should evaluate rituximab-based steroid-sparing regimens for the management of AAV with severe renal involvement.

## INTRODUCTION

The antineutrophil cytoplasmic antibody (ANCA)-associated vasculitides (AAV) are a rare group of autoimmune inflammatory conditions affecting small to medium blood vessels, largely comprised of granulomatosis with polyangiitis and microscopic polyangiitis [1, 2]. AAV can present with symptoms affecting various organs, but commonly affects the kidneys, lungs, upper airways and skin [3]. Renal involvement in AAV occurs in over 75% of patients [4].

Renal presentation of AAV is associated with increased morbidity and mortality [5], especially when prompt treatment is delayed [6]. Standard treatment in life-threatening AAV is a combination of glucocorticoids, together with cyclophosphamide (CYC) or rituximab (RTX) [7]. Prior to the use of RTX, CYC was the mainstay of treatment for AAV [7, 8]. The RAVE (Rituximab versus Cyclophosphamide for ANCA-Associated Vasculitis) and RITUXIVAS (Rituximab versus Cyclophosphamide in ANCA-Associated Renal Vasculitis) trials showed RTX to be non-inferior to CYC. Recent guidelines recommend either CYC or RTX, together with steroids with or without plasma exchange, for induction of remission in AAV [9]. Given the higher risk of leukopenia and severe infections with CYC, RTX has become first line for induction therapy in many centres [10–12]. However, there were limitations associated with both the RAVE and RITUXIVAS landmark studies. RAVE excluded patients with severe renal involvement, and whilst RITUXIVAS accounted for these patients, participants in RITUXIVAS also received two pulses of intravenous CYC together with RTX.

Further observational studies have since addressed the paucity of data when comparing CYC and RTX in AAV with severe renal impairment. Geetha *et al*. [13] studied 37 patients who received RTX, or RTX and CYC, with a presenting estimated glomerular filtration rate (eGFR) <20 mL/min/1.73 m^2^, and showed equivalent outcomes. However, they did not independently compare solely CYC with RTX. More recently, Moura *et al*. [14] performed a larger retrospective analysis between 1996 and 2015 of 251 patients with severe renal impairment, defined as eGFR <30 mL/min/1.73 m^2^, comparing remission-induction outcomes in response to RTX compared with CYC. The mean eGFR for the two groups was 17.5 mL/min/1.73 m^2^ in this study, which found no significant difference between CYC and RTX for the treatment of renal impairment in AAV. However, the number of patients requiring dialysis at the time of presentation was relatively low (15%).

It remains unclear whether RTX is as efficacious as CYC at inducing remission for patients with severe renal involvement, including those who present dialysis dependent. We therefore aimed to assess remission induction outcomes between RTX- and CYC-treated patients, with severe renal impairment defined as serum creatinine >354 µmol/L, or dialysis dependency, at presentation.

## MATERIALS AND METHODS

A tertiary centre, retrospective cohort study was conducted to assess the outcomes of RTX compared with CYC as an induction agent, in combination with high-dose steroids and with or without plasma exchange, in patients with a *de novo* presentation of AAV with severe renal involvement. The study included patients who presented to Queen Elizabeth Hospital Birmingham and Birmingham Heartlands Hospital between 2010 and 2023. All consecutive patients aged >18 years with severe renal impairment defined as serum creatinine >354 µmol/L with a clinical presentation of AAV or biopsy-proven AAV were included in the study. This was a quality improvement project (CARMS audit number: CARMS-21610) to evaluate our regional induction of remission protocol, and therefore data were collected as part of clinical care.

Data was collated from electronic patient records systems and included demographics, baseline disease, biopsy data using the ANCA Kidney Risk Score score [15], treatment parameters and outcome data. Infections that required the use of intravenous antibiotics or hospital admission were classed as severe infections.

The primary outcome was sustained remission with independent renal function at 12 months. Sustained remission at 12 months was defined as the complete absence of clinical disease activity, with no new or worsening organ involvement, stable or improving organ function, and no requirement for escalation of immunosuppressive therapy for at least 6 consecutive months. Independent renal function at 12 months was defined as patient survival with native kidney function sufficient to avoid dialysis during the preceding 90 days and without having received a kidney transplant. Secondary outcomes comprised of renal function at 3, 6 and 12 months (eGFR was calculated via the Chronic Kidney Disease Epidemiology Collaboration equation), independent renal function at 3 and 12 months, survival at 3 and 12 months, relapse within 12 months, infections within 12 months and tolerability of induction of remission therapy. Relapse was defined as the reappearance of clinical disease activity attributable to ANCA-associated vasculitis after a period of remission, characterized by new or worsening organ involvement and requiring escalation of immunosuppressive therapy.

Data were analysed using SPSS Version 30 (IBM Corp., Armonk, NY, USA) and GraphPad Prism Version 10 (GraphPad Software, San Diego, CA, USA). Baseline characteristics, primary outcome and renal function between CYC and RTX were compared using the Chi-square test (or Fisher’s exact test where expected cell counts were low) and Mann–Whitney U test. Kaplan–Meir survival curve analysis with log-rank test for group comparisons was used to assess differences in time to relapse, time to infection and survival rates. To account specifically for the competing risk of mortality when assessing time to first relapse, a Fine–Gray subdistribution hazard model was employed. This model treats death as a competing event rather than a standard censoring event, providing a more accurate estimation of relapse probability in this cohort. Binary logistic regression was performed to identify independent predictors of the primary composite outcome at 12 months. The multivariable model included the following covariates: age, ANCA serotype [myeloperoxidase (MPO) vs proteinase 3 (PR3)], use of plasma exchange (PLEX), treatment type (RTX vs CYC) and dialysis dependence at baseline. Odds ratios (OR) with 95% confidence intervals (CI) were calculated. A secondary analysis was conducted to exclude patients who had been treated with both CYC and RTX at induction, due to CYC intolerance, to solely assess the impact of either agent. To further address potential confounding by indication and treatment era imbalances—specifically regarding dialysis dependence, PLEX use, and intravenous methylprednisolone (MP) exposure—a propensity score matching analysis was performed. Propensity scores were estimated using binary logistic regression, and patients were matched using a nearest-neighbour approach without replacement. Post-matching balance was verified by comparing key baseline variables between groups.

## RESULTS

### Baseline and treatment characteristics

A total of 107 patients presenting with AAV and severe renal involvement that fulfilled the inclusion criteria were included in this analysis. Sixty-six patients received 6–10 pulses of intravenous CYC (median cumulative dose 5.6 g), and 41 patients received RTX at a dose of 1 g at baseline followed by 1 g at Week 2, together with high-dose glucocorticoids for remission induction. Baseline characteristics were well balanced between the two cohorts (Table 1), with no significant differences in median age, sex or ANCA subtype. There was a similar proportion of patients with extra-renal disease with 41% in each group presenting with additional manifestations, including pulmonary haemorrhage, lung involvement, neurological involvement or ear–nose–throat involvement, with no statistically significant differences between the types of extra-renal involvement in RTX- versus CYC-treated patients. The CYC-treated cohort had significantly greater use of PLEX and MP pulses (PLEX: 59% of CYC compared with 17% of RTX patients, *P* < .001; MP: 53% of CYC compared with 27% of RTX patients, *P* = .008). The oral glucocorticoid protocol is outlined in Table 2. No patients received avacopan. For Pneumocystis jirovecii pneumonia (PCP) prophylaxis a 6-month regimen of 480 mg co-trimoxazole on alternate days was given. No patients received intravenous immunoglobulin.

**Table 1:** Baseline and treatment characteristics.

	Total	CYC	RTX	*P*-value
N	107	66	41	
Men/women	62/45	42/24	20/21	.130
Age (years)	70 (61–76)	70 (61–76)	70 (59–76)	.780
ANCA +ve	98	63 (95)	35 (85)	.068
ANCA −ve	9	3	6	
MPO	50	34 (54)	16 (46)	.434
PR3	48	29 (46)	19 (54)	
Extra-renal disease	44	27 (41)	17 (41)	.955
Pulmonary haemorrhage	14	8 (30)	6 (35)	.708
Lung involvement	17	9 (33)	8 (47)	.419
Neurological involvement	4	3 (11)	1 (6)	.577
ENT	18	14 (52)	4 (24)	.124
Treatment				
PLEX	46	39 (59)	7 (17)	<.001
MP	46	35 (53)	11 (27)	.008
Dose of treatment (g)		5.6 (3.3–7.8)	2 (2–2)	

Median (interquartile range) shown, or *n* (%). ENT, ear–nose–throat.

**Table 2:** Oral glucocorticoid taper regimen.

Week 1	60 mg once daily
Week 2	40 mg once daily
Week 3–4	30 mg once daily
Week 5–6	25 mg once daily
Week 7–8	20 mg once daily
Week 9–10	15 mg once daily
Week 11–12	15 mg and 10 mg on alternate days
Week 13–14	10 mg once daily
Week 15–16	10 mg and 5 mg on alternate days
Week 17	5 mg once daily

At baseline, similar numbers of CYC-treated patients compared with RTX-treated patients required renal replacement therapy, including those with severe but potentially rapidly reversible acute kidney injury at presentation (56% vs 71%, *P* = .129) (Table 3). Similarly, there was no difference in presenting renal function between the two groups. Presenting eGFR was 9 (6–11) mL/min/1.73 m^2^ in the CYC group and 7 (6–10) mL/min/1.73 m^2^ in the RTX group (*P* = .372).

**Table 3:** Renal function at baseline, 3, 6 and 12 months.

	CYC		RTX		*P*-value
	***n***		***n***		
Creatinine (µmol/L), median (IQR)					
Baseline	66	511 (422–655)	41	538 (487–643)	.399
12 month	38	186 (121–247)	17	168 (126–211)	.362
ACR (mg/mmol), median (IQR)					
Baseline	46	175 (110–309)	35	168 (78–359)	.823
12 month	47	49 (21–103)	22	37 (9–72)	.395
Dialysis dependence, *n* (%)					
Baseline	66	37 (56)	41	29 (71)	.129
3 month	65	24 (37)	38	18 (47)	.298
6 month	63	25 (39)	36	15 (42)	.799
12 month	62	22 (35)	32	14 (44)	.582
eGFR (mL/min/1.73 m^2^), median (IQR)					
Baseline	63	9 (6–11)	41	7 (6–10)	.372
3 month	38	24 (18–36)	17	28 (22–36)	.291
6 month	36	29 (19–36)	20	29 (21–35)	.884
12 month	37	28 (21–40)	17	31 (28–49)	.439

IQR, interquartile range.

A renal biopsy was performed in 103 of 107 patients. There was no difference in the proportion of normal, sclerosed or crescentic glomeruli at the baseline biopsy between CYC- and RTX-treated patients (Table 4). ANCA renal risk score analysis showed a similar proportion of low, moderate, high and very high risk scores in both CYC- and RTX-treated patients (*P* = .077). (Table 4).

**Table 4:** Renal baseline biopsy data.

	CYC	RTX	Total	*P*-value
No. of biopsies	64	39	103	
Normal glomeruli (%)	23	24		.708
Crescentic glomeruli (%)	56	49		.141
Globally sclerosed glomeruli (%)	16	21		.147
Chronic damage (%)	15	18		.480
AKRiS, *n* (%)				.077
Low	4 (6)	6 (15)	10 (10)	
Moderate	28 (44)	11 (28)	39 (38)	
High	27 (42)	14 (36)	41 (40)	
Very high	5 (8)	8 (21)	13 (13)	

AKRiS, ANCA Kidney Risk Score.

Following induction of remission, patients that recovered independent renal function received maintenance of remission therapy. The majority of CYC-treated patients received maintenance therapy with azathioprine. RTX-treated patients that received maintenance therapy were treated with further RTX infusions or azathioprine with a small minority of patients receiving mycophenolate mofetil (Table 5).

**Table 5:** Maintenance therapy following induction of remission.

	AZA	RTX	MMF	None^a^
CYC	39 (59)	8 (12)	2 (3)	14 (21)
RTX	7 (17)	10 (24)	2 (5)	12 (29)

Date are presented as *n* (%). ^a^These patients remained dialysis dependent. AZA, azathioprine; MMF, mycophenolate mofetil.

### Primary outcome

The primary outcome, sustained remission with independent renal function at 12 months was achieved in 20/41 (49%) of the RTX cohort and 34/66 (52%) of the CYC cohort (*P* = .783).

**Figure 1:**
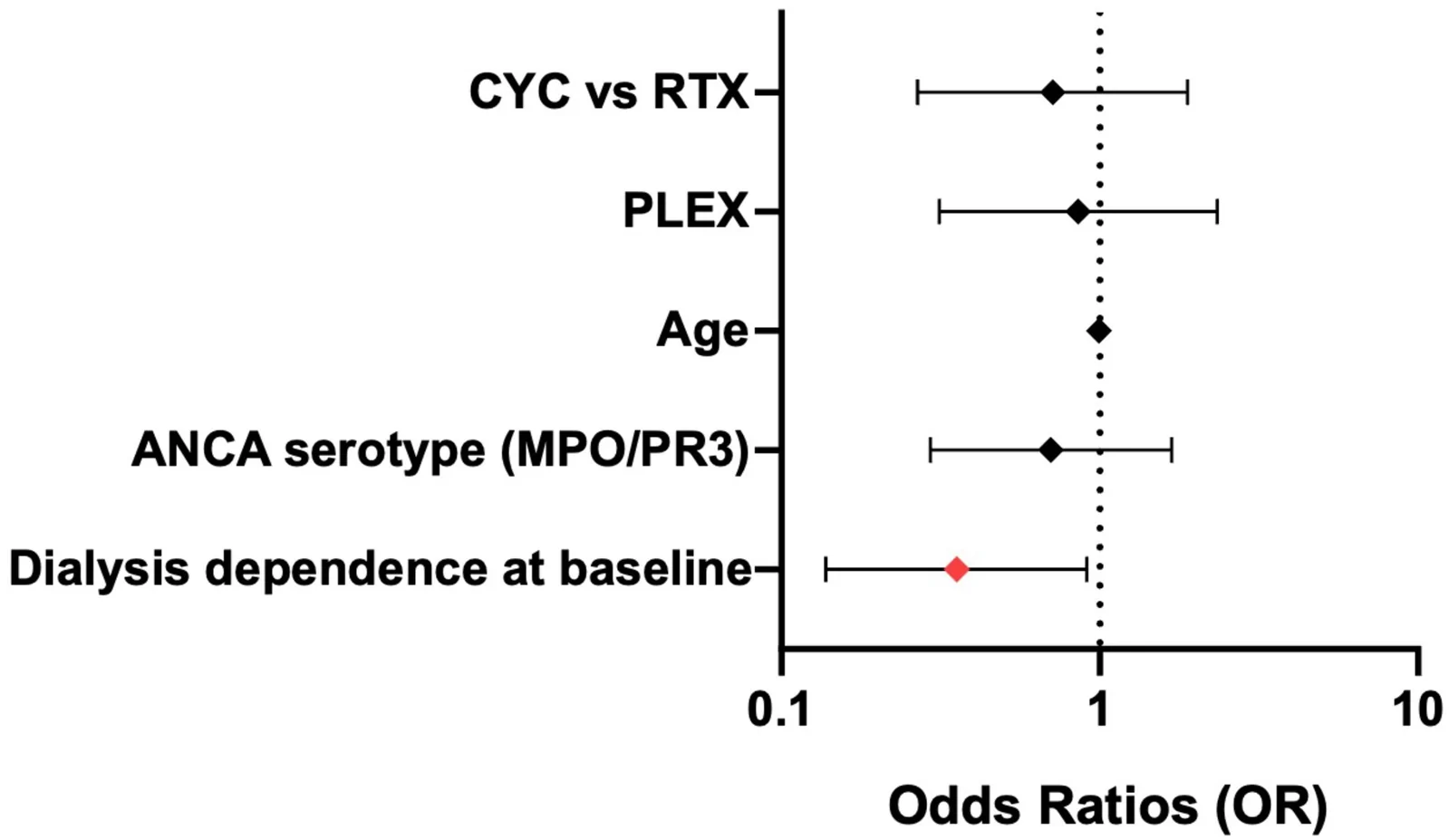
Clinical parameters associated with sustained remission with independent renal function at 12 months. Odds ratio (95% CI) is displayed for induction treatment CYC vs RTX, PLEX, age, ANCA serotype (MPO/PR3) and dialysis dependence at baseline. The red diamond depicts a statistically significant difference; OR (CI) 0.355 (0.138–0.912); *P *= .031.

We conducted a multivariable analysis to understand the main factors associated with failing to achieve the primary outcome (Fig. 1). This revealed that dialysis dependency at presentation was a strong independent predictor of not attaining the primary outcome at 12 months [odds ratio (CI) 0.355 (0.138–0.912), *P *= .031]. Type of induction treatment used (CYC vs RTX) did not have a statistically significant impact on the primary outcome (*P *= .495). Similarly, ANCA serotype (MPO versus PR3), age or use of PLEX therapy did not affect the primary outcome (*P* = .426, .778 and .763, respectively).

### Secondary outcomes

There were no statistically significant differences when comparing independent renal function between CYC- and RTX-treated patients at 3 months (63% vs 53%, *P* = .298), 6 months (61% vs 58%, *P* = .799) or 12 months (65% vs 56%, *P* = .582) (Table 3).

**Figure 2:**
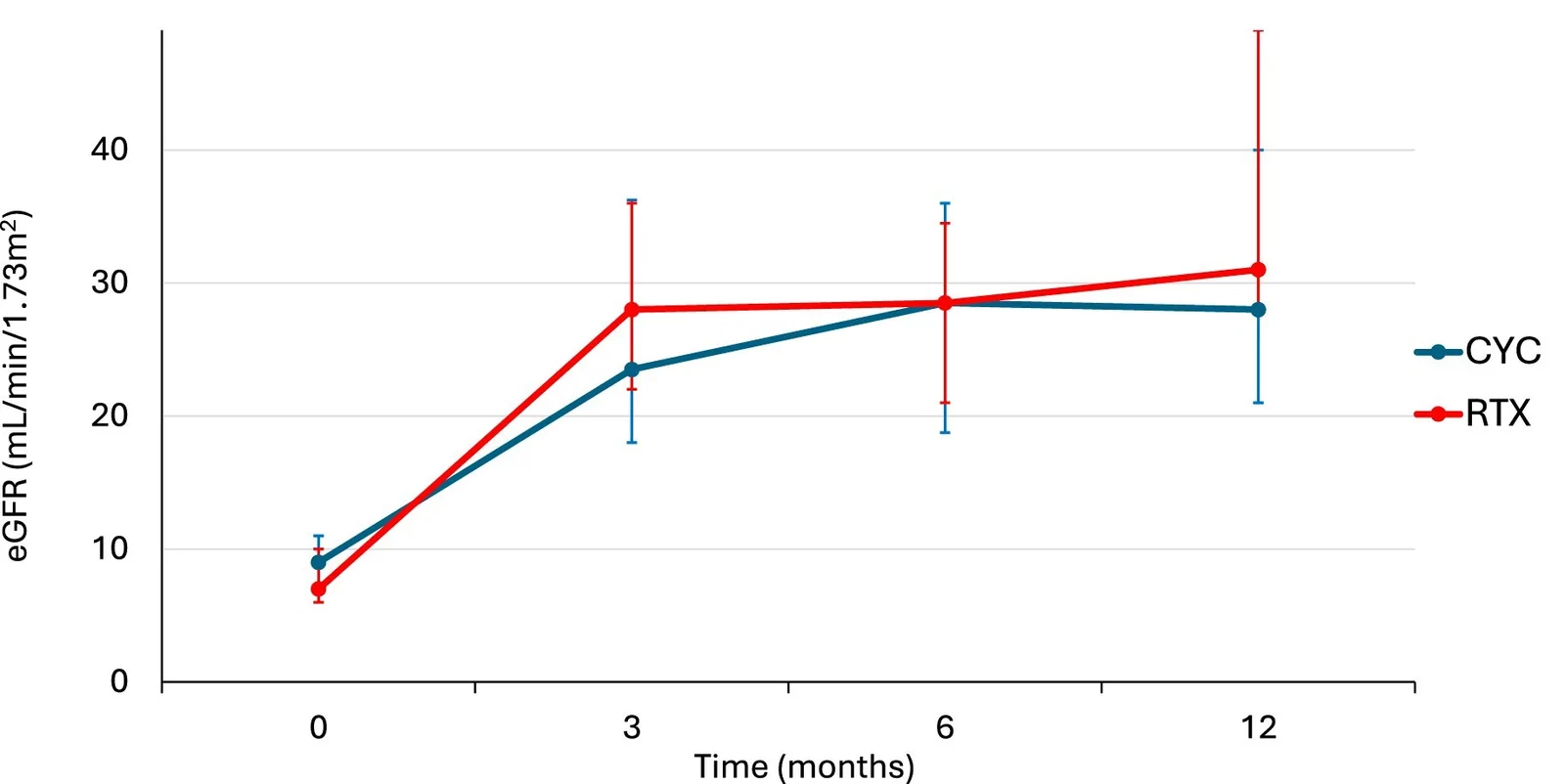
Change in eGFR from baseline to 12 months in CYC- versus RTX-treated patients. The blue line depicts CYC-treated patients and the red line depicts RTX-treated patients (median ± interquartile range). A sustained increase in eGFR from baseline was seen in both treatment arms but there was no statistically significant difference at baseline, 3, 6 or 12 months between CYC- and RTX-treated patients.

Similarly, there were no significant differences in eGFR at 3, 6 or 12 months (Fig. 2). Longitudinal eGFR analyses from 3 months onwards included only patients who had recovered renal function, with patients remaining dialysis-dependent at each time point excluded from these analyses. At 3 months, eGFR was 24 (18–36) mL/min/1.73 m^2^ in the CYC group and 28 (22–36) mL/min/1.73 m^2^ in the RTX group (*P* = .291). At 6 months, eGFR was 29 (19–36) mL/min/1.73 m^2^ in the CYC group and 29 (21–35) mL/min/1.73 m^2^ in the RTX group (*P* = .884). At 12 months, eGFR was 28 (21–40) mL/min/1.73 m^2^ in the CYC group and 31 (28–49) mL/min/1.73 m^2^ in the RTX group (*P* = .439).

There were no statistically significant differences in urine albumin–creatinine ratio (ACR) measurements at baseline and 12 months between CYC- and RTX-treated patients. At baseline urine ACR was 175 (101–309) mg/mmol in the CYC group compared with 168 (978–359) mg/mmol in the RTX group (*P* = .823). At 12 months, urine ACR was 49 (21–103) mg/mmol in the CYC group compared with 37 (9–72) mg/mmol in the RTX group (*P* = .395).

Two patients who had independent renal function and were treated with CYC at presentation became dialysis dependent by 12 months, whereas no RTX-treated patients with independent renal function at baseline became dialysis dependent (*P *= .351). Forty-one percent (*n* = 15) of CYC dialysis-dependent patients at baseline regained independent renal function by 12 months at a median of 3 (3–173) days, compared with 31% (*n* = 9) of RTX-treated dialysis-dependent patients at baseline who regained independent renal function by 12 months at a median of 30 (3–59) days (*P *= .426).

PLEX and intravenous pulsed MP use did not affect dialysis dependence at 12 months (*P *= .625 and .234, respectively).

**Figure 3:**
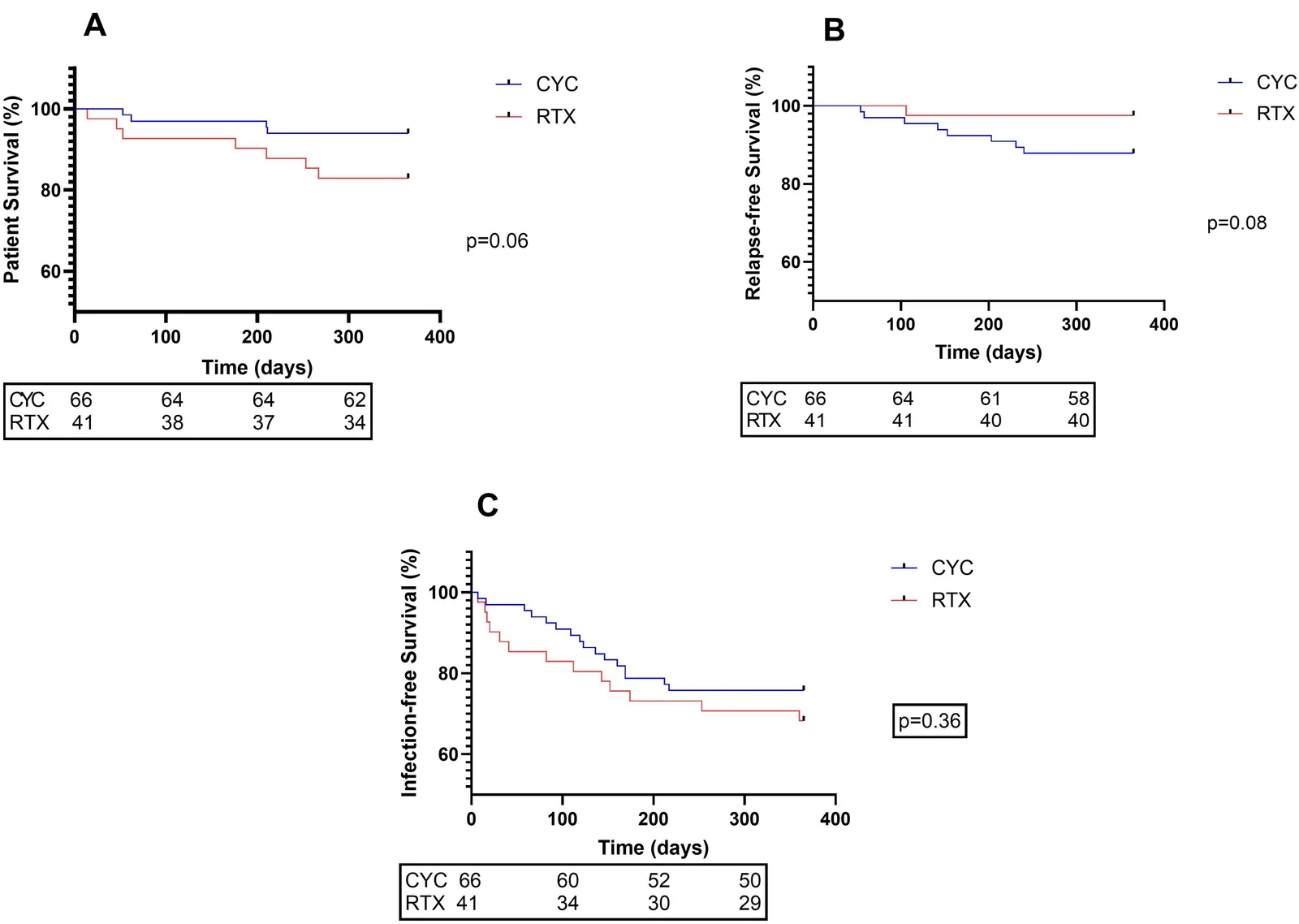
Overall survival, relapses and infections. Time to death (**A**), time to first relapse (**B**) and time to first severe infection (**C**) by 12 months is shown in blue for patients who received CYC and red for patients who received RTX. The table below the graphs shows the number of patients alive and therefore at risk at each given time point.

When assessing survival (Fig. 3A), 94% of CYC patients compared with 83% of RTX patients were alive at 12 months (*P* = .068). Three of the seven deaths in the RTX cohort were secondary to COVID-19, reflecting the much greater use of RTX during the COVID-19 pandemic in our centre. No deaths in the CYC cohort were secondary to COVID-19. A further three deaths in the RTX cohort were associated with severe pre-existing comorbidities.

When assessing relapses within 12 months from induction of remission (Fig. 3B), eight CYC patients (12.1%) relapsed compared with one RTX patient (2.4%), however this difference was not statistically significant (*P* = .079). Five of the eight relapses in the CYC cohort happened within 6 months. The RTX relapse also occurred within 6 months.

**Figure 4:**
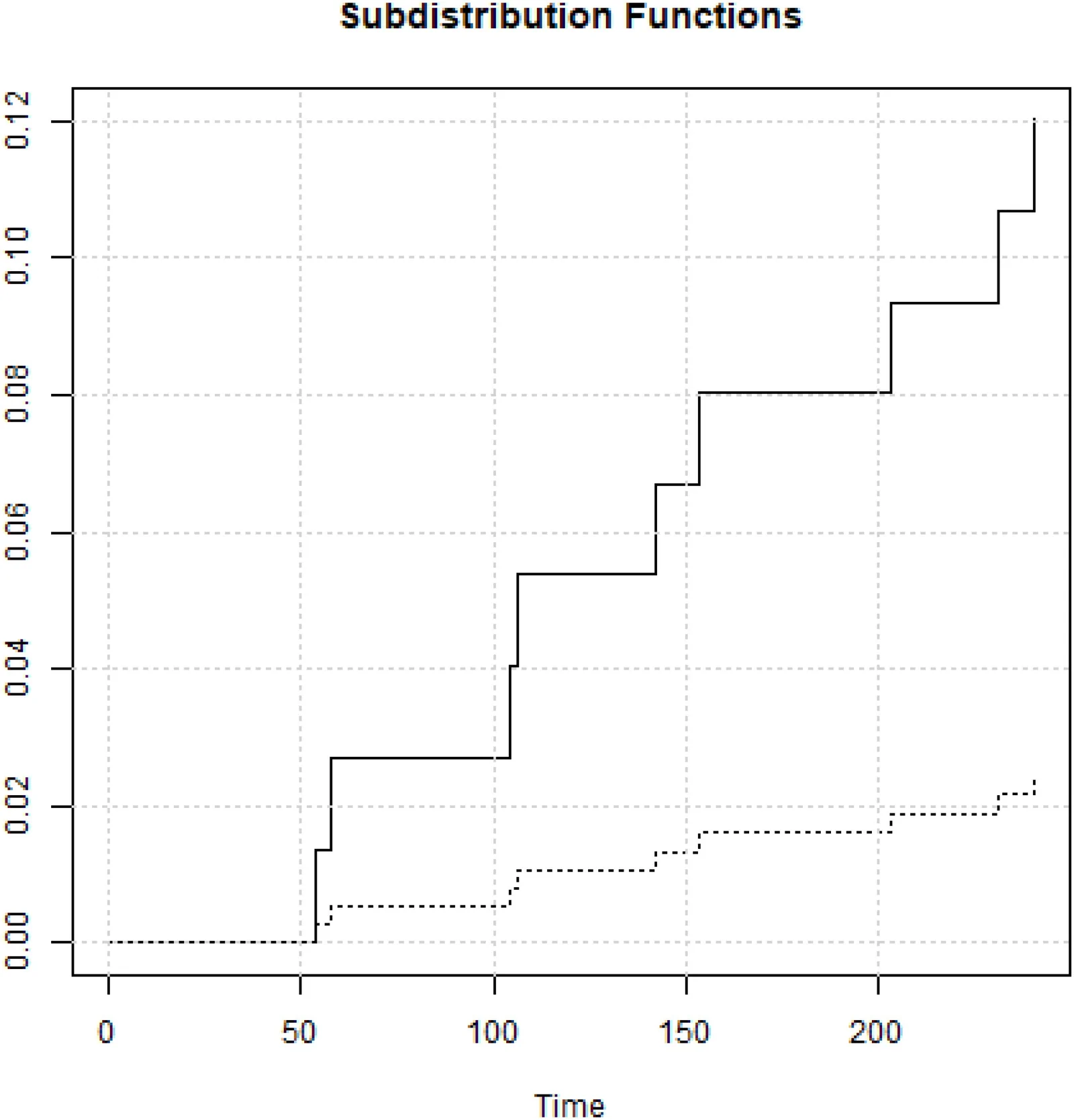
Cumulative incidence of relapse accounting for the competing risk of death. Cumulative incidence function (CIF) curves for first relapse in patients treated with RTX (dotted line) versus CYC (solid line). Death was treated as a competing risk using the Fine–Gray method. The curves represent the probability of experiencing a relapse over 12 months while accounting for mortality as a competing event.

To account for the numerically higher mortality observed in the RTX group, a competing risk analysis was performed using the Fine–Gray subdistribution hazard model, treating death as a competing event for relapse. This analysis confirmed that the lower risk of relapse in the RTX group remained consistent even after accounting for the competing risk of death [subdistribution hazard ratio (sHR) 0.193; 95% CI, 0.024–1.544; *P* = .120]. While the difference was not statistically significant, the lower sHR supports the primary finding of a numerically reduced relapse risk in patients treated with RTX compared with CYC (Fig. 4).

When assessing infection rates (Fig. 3C), 24.2% of CYC patients (*n* = 16) compared with 31.7% of RTX patients (*n* = 13) developed severe infections within 12 months of induction (*P* = .398).

### Tolerability

Seven (11%) CYC patients had tolerability issues with CYC compared with none with RTX (*P* = .31). Three patients developed cytopenia, two developed rashes and one patient had visible haematuria. Patients who were intolerant to CYC were treated with RTX.

### Secondary analyses

A secondary analysis was conducted by excluding the seven patients who received both CYC and RTX at induction due to tolerability issues with CYC, to assess the impact of CYC alone compared with RTX alone. When re-assessing the primary outcome, 56% CYC compared with 49% RTX were in sustained remission with independent renal function at 12 months (*P* = .481). Secondary analysis of survival and relapse events at 12 months also did not reveal any statistically significant differences between the CYC and RTX groups (*P* = .106 and *P* = .087).

A propensity scored–matched sensitivity analysis was conducted to account for induction of remission treatment differences between the two groups, namely use of PLEX and intravenous MP (Table 1). Following propensity score matching, a well-balanced cohort of 30 pairs was identified with no significant differences in dialysis dependence (57% vs 60%, *P *= .80), PLEX use (23% vs 23%, *P *= 1.00) or intravenous MP exposure (46% vs 36%, *P *= .43). Within this matched cohort, the primary composite outcome of sustained remission with independent renal function was achieved in 16 patients (53%) in both the CYC and RTX groups (*P *= 1.00). These findings are consistent with the primary unmatched analysis, further supporting the comparable efficacy of the two induction strategies (Table 6).

**Table 6:** Baseline parameters and outcome of the propensity score matched sensitivity analysis.

Characteristic	CYC (*n* = 30)	RTX (*n* = 30)	*P*-value
Demographics			
Age, median (IQR)	75 (69–79)	70 (58–76)	.021
Female, *n* (%)	13 (43)	13 (43)	1
AKRiS			.22
Low	4	4	
Moderate	11	8	
High	13	10	
Very high	1	6	
Dialysis dependence, *n* (%)	17 (57)	18 (60)	.8
ANCA serotype (PR3), *n* (%)	12 (41)	15(50)	.17
Treatment parameters, *n* (%)			
PLEX use	7 (23)	7 (23)	1
IV MP pulse	14 (46)	11 (36)	.43
Primary outcome, *n* (%)			
Sustained remission at 12 months	16 (53)	16 (53)	1

AKRiS, ANCA Kidney Risk Score; IQR, interquartile range.

## DISCUSSION

In this retrospective analysis of 107 patients with AAV and severe renal involvement we show that RTX is comparable to CYC together with high-dose steroids to induce remission. This is the largest cohort comparing outcomes between CYC- and RTX-treated patients with severe renal disease. It should be noted that 62% of patients in our study were dialysis dependent at presentation, with a median presenting eGFR of 8 mL/min/1.73 m^2^ across the cohort. Importantly, there was no difference in rates of renal replacement therapy recovery, renal function or proteinuria between CYC- and RTX-treated patients at 3, 6 or 12 months. Our findings suggest that induction of remission with RTX achieves comparable renal function recovery compared with CYC at these critical early time points which persists to Month 12. This is important as renal function and disease activity at 3 and 6 months are predictive of the long-term risk of mortality and end-stage renal disease in AAV [16].

The patient cohorts were well balanced with respect to gender, age, ANCA (MPO/PR3) and extra-renal disease at presentation, with a similar proportion of acutely unwell patients presenting with pulmonary haemorrhage. Both patient groups had similar renal biopsy data as evidenced by the percentage of normal, crescentic and globally sclerosed glomeruli. ANCA renal risk scores were comparable between the two treatment cohorts. Baseline renal function was also comparable between both cohorts, with no significant difference in presenting eGFR or requirement for renal replacement therapy at presentation. Notably both groups had a median eGFR <10 mL/min/1.73 m^2^ and median creatinine of >500 µmol/L at presentation, representing a population with severe renal involvement.

Renal function improved the most within the first 3 months of starting induction with either RTX (median eGFR increase of +21 mL/min/1.73 m^2^) or CYC (+15 mL/min/1.73 m^2^). This mirrors similar findings in other studies [17]. Sixty-five percent of CYC patients and 56% of RTX patients had independent renal function at 12 months. These rates of renal recovery are comparable to those shown in other studies evaluating outcomes amongst patients with AAV presenting with severe renal involvement. Moura *et al*. [18] reported independent renal function in 66.7% of patients presenting with eGFR <15 mL/min/1.73 m^2^ or requiring dialysis, whilst in another study investigating outcomes for patients presenting with severe renal involvement defined as creatinine >500 µmol/L or dialysis dependency, recovery of independent renal function occurred in 57.7%–66.1% [19]. Moura *et al*. found predictors of end stage renal disease at 12 months to be related to a higher serum creatinine at diagnosis and moderate to severe chronicity changes in the renal biopsy [18]. Similarly, in a multivariable analysis we found that the only predictor of the primary outcome, sustained remission with independent renal function at 12 months, was dialysis dependency at presentation. Age has previously been shown to not be a factor for dialysis dependence [20], and this is mirrored in our findings here.

We observed numerically more deaths in the RTX- compared with CYC-treated patients, although this difference was not statistically significant. During the COVID-19 pandemic we started using RTX almost exclusively to induce remission in order to reduce footfall in the infusion suites. Consequently, several of the deaths recorded with RTX therapy were secondary to COVID-19. Once accounting for the COVID-19 deaths, there were four deaths in each cohort. Similarly other studies, including a large cohort of 595 vasculitis patients, found there to be no difference in mortality or end-stage renal disease between RTX or CYC [21].

The rate of infections requiring hospital admission or intravenous antibiotics was comparable between the two cohorts of CYC and RTX. Similar infection rates were noted in a *post hoc* analysis of the RAVE trial [22], suggesting that the infection risk is predominantly driven by high-dose corticosteroid treatment as previously demonstrated [19, 20].

Although more relapses occurred with CYC compared with RTX this was not statistically significant. RAVE and RITUXIVAS also did not find a significant difference in the rate of relapses within 12 months [10, 11]. Five relapses occurred within the first 6 months in the CYC cohort, compared with one in the RTX cohort. These early relapses are likely to be related to the induction agent. Three patients relapsed after 6 months in the CYC cohort compared with none in the RTX cohort; it is likely that these later relapses reflect differences in maintenance therapy rather than induction agent, as azathioprine is associated with greater relapse risk than RTX when used for maintenance.

With respect to adverse events and tolerability of therapy, although there were more tolerability issues with CYC, this difference was not statistically significant, a finding that is also congruent with that of RAVE and RITUXIVAS.

Our study has limitations. It is a retrospective analysis and therefore there may be additional confounding factors that we have not accounted for. Due to a change in treatment protocol around the COVID-19 pandemic, patients treated with CYC represent a predominantly earlier cohort compared with those treated with RTX. However, baseline characteristics including severity were well balanced between the two groups. The observed increased MP and plasma exchange use with CYC is likely driven by the time-trend bias of more CYC use in the earlier years of this study. MP pulses have been shown to not affect survival, renal recovery or relapses [18]. A meta-analysis of the utility of PLEX therapy showed benefit in that use of PLEX reduced rates of end stage renal disease at 12 months [20]. It is possible that the substantially reduced use of PLEX in the RTX-treated patients may have masked a potential benefit of PLEX in this group. In addition, we analysed solely RTX and high-dose corticosteroids with solely CYC with high-dose corticosteroids. We did not assess potential benefit of a combined pulse CYC and RTX strategy as previously reported by McAdoo *et al*. [23].

## CONCLUSION

In conclusion, in this cohort of 107 patients with AAV and severe renal involvement with serum creatinine >354 µmol/L or dialysis dependency at presentation, we find that induction of remission with RTX in combination with high-dose steroids is comparable to pulsed CYC with high-dose steroids. Future studies should investigate whether rituximab-based steroid-sparing therapeutic regimens can achieve comparable outcomes with reduced glucocorticoid exposure in patients with severe renal involvement. 

## Data Availability

The data underlying this article cannot be shared publicly due to patient privacy and NHS information governance. De-identified data may be available from the corresponding author upon reasonable request and with appropriate approvals.
